# Progress in Transcriptomics and Metabolomics in Plant Responses to Abiotic Stresses

**DOI:** 10.3390/cimb47060421

**Published:** 2025-06-05

**Authors:** Tao Yu, Xuena Ma, Jianguo Zhang, Shiliang Cao, Wenyue Li, Gengbin Yang, Changan He

**Affiliations:** 1 Maize Research Institute of Heilongjiang Academy of Agricultural Sciences, Harbin 150086, Chinacaoshiliang2003@126.com (S.C.); liwenyue199211@163.com (W.L.); kshmaize@163.com (G.Y.); 2 Key Laboratory of Biology and Genetics Improvement of Maize in Northern Northeast Region, Ministry of Agriculture and Rural Affairs, Harbin 150086, China; 3 Key Laboratory of Germplasm Resources Creation and Utilization of Maize, Harbin 150086, China; 4 Keshan Branch of Heilongjiang Academy of Agricultural Sciences, Qiqihaer 161000, China; corn_he@163.com

**Keywords:** plants, abiotic stress, transcriptomics, metabolomics, progress

## Abstract

Abiotic stress constrains plant growth and productivity worldwide. To survive adverse environmental conditions, plants deploy sophisticated adaptive strategies involving transcriptional reprogramming and metabolic remodeling. Over the past decade, advancements in high-throughput sequencing and mass spectrometry have propelled transcriptomics and metabolomics as pivotal post-genomic disciplines, offering unprecedented opportunities to dissect molecular mechanisms underlying stress responses. This review synthesizes current progress in applying these omics technologies to investigate plant adaptations to key abiotic stresses (thermal, saline, water deficit/excess, and heavy metal stresses). We systematically evaluate the technical strengths and limitations of transcriptomic and metabolomic platforms, highlight recent breakthroughs in stress-responsive gene identification and metabolic pathway elucidation, and discuss emerging challenges in integrative data analysis. By bridging genotype–phenotype relationships through multi-omics approaches, this study aims to deepen our mechanistic understanding of plant stress resilience and inform the design of stress-resilient crops for sustainable agriculture.

## 1. Introduction

In the post-genomic era, transcriptomics and metabolomics have emerged as key pillars of systems biology, complementing genomics and proteomics to provide multi-dimensional insights into biological processes [1]. Transcriptomic profiling enables the quantitative analysis of genome-wide mRNA expression dynamics under stress conditions, allowing researchers to dissect complex regulatory networks underlying plant stress adaptation and identify candidate resistance genes. Beyond differential expression analysis, this technology has proven indispensable for transcriptional network reconstruction, gene family characterization, and the discovery of functional markers through polymorphism detection [2]. Conversely, metabolomics focuses on the comprehensive profiling of low-molecular-weight metabolites (<1 kDa) across biological systems, serving as a bridge between genotype and phenotype. Recent advancements in mass spectrometry-based platforms now enable an unbiased detection of diverse metabolite classes, providing critical insights into metabolic reprogramming during abiotic stress responses [3]. Importantly, the integration of multi-omics approaches—spanning genomics, epigenomics, proteomics, and lipidomics—has revolutionized our capacity to decode plant stress adaptation mechanisms. For instance, proteogenomic analyses combining transcriptome and proteome data have uncovered post-translational modifications that regulate drought-responsive signaling cascades in maize [4], while integrated epigenome-transcriptome studies have revealed DNA methylation patterns modulating heat-responsive gene expression in rice [5]. Lipidomic–metabolomic integration further demonstrated membrane lipid remodeling as a conserved strategy under combined cold and salinity stress [6]. Such multi-omics frameworks not only resolve the temporal–spatial dynamics of stress responses but also elucidate cross-talk between transcriptional regulation and metabolic homeostasis [7]. Collectively, these omics approaches have been instrumental in deciphering stress tolerance mechanisms in major crops, including maize, rice, and soybean [7,8,9,10].

The intensification of global climate change has exacerbated the frequency and severity of abiotic stressors, posing unprecedented challenges to agricultural productivity and food security [11]. Rising temperatures, erratic precipitation patterns, and soil degradation have amplified the impacts of drought, salinity, and heavy metal contamination, with projections indicating potential yield losses of 10–50% for staple crops by 2050 [12]. Abiotic stressors, encompassing temperature extremes, salinity, drought, waterlogging, and heavy metal toxicity, disrupt plant homeostasis and often act synergistically to compound their detrimental effects on growth and development [13,14,15]. To mitigate these impacts, plants have evolved sophisticated sensory and adaptive systems that integrate transcriptional and metabolic reprogramming to maintain cellular integrity and energy balance [12].

Recent breakthroughs in multi-omics technologies, such as single-cell RNA sequencing (scRNA-seq) and spatially resolved metabolomics, now allow for unprecedented resolution in mapping cell type-specific responses and tissue-level metabolic heterogeneity under stress [16,17]. For example, scRNA-seq has revealed distinct transcriptional trajectories in root epidermal cells under salt stress [18], while spatial metabolomics have uncovered plant metabolic responses to water-deficit stress [19]. Leveraging these technologies, studies have identified conserved regulatory modules (e.g., ABA-dependent signaling hubs) and metabolic signatures (e.g., flavonoid biosynthesis) associated with stress resilience across species [8]. Furthermore, systems biology approaches integrating genome-wide association studies (GWASs) with metabolome-wide association studies (MWASs) have accelerated the discovery of genetic loci controlling stress-responsive metabolites, enabling the marker-assisted breeding of resilient cultivars [20]. This review systematically synthesizes current progress in applying transcriptomic and metabolomic technologies to decipher plant adaptations to abiotic stresses and evaluates their technical capabilities and analytical challenges in identifying stress-responsive genes and metabolic networks; ultimately, it aims to provide a basis for the rational design of stress-resistant crops in sustainable agriculture.

## 2. Transcriptomics

Transcriptomics encompasses the global analysis of gene transcription and regulatory networks in biological systems. This discipline was pioneered by Velculescu et al. in 1997, marking the first systematic profiling of cellular mRNA populations [21]. Broadly defined, the transcriptome represents the complete set of RNAs—including messenger (mRNA), ribosomal (rRNA), transfer (tRNA), and non-coding (ncRNA) species—expressed under specific physiological or environmental conditions. Narrowly interpreted, it refers to the protein-coding mRNA fraction, which remains the primary focus of most transcriptomic studies. By quantifying gene expression dynamics at the transcriptional level, transcriptomics uncovers molecular mechanisms underlying biological processes, ranging from developmental programs to stress responses. Since its inception, technological advancements have solidified transcriptomics as a cornerstone of modern biological research [22,23].

### 2.1. Comparative Analysis of Transcriptomic Research Technologies

**Figure 1:**
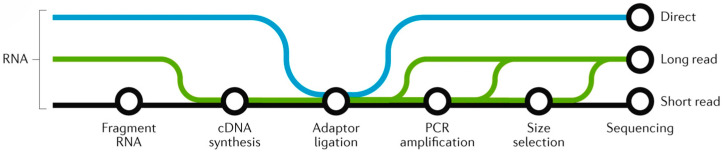
The workflow of RNA sequencing.

Transcriptomic technologies can be broadly classified into two categories based on their underlying principles, namely hybridization-based methods (e.g., DNA microarrays) and sequencing-based approaches (e.g., RNA-seq). DNA microarrays utilize fluorescently labeled cDNA probes to measure relative gene expression by comparing signal intensities between experimental and control samples, with red and green dyes typically used for differential labeling. Sequencing-based platforms include EST, SAGE, MPSS, RNA-seq, and single-cell RNA-seq (scRNA-seq). Among these, RNA-seq has emerged as the gold standard due to its high throughput, single-nucleotide resolution, and ability to detect novel transcripts and alternative splicing events. The workflow involves RNA fragmentation, cDNA synthesis, adapter ligation, PCR enrichment, and high-throughput sequencing, followed by a bioinformatics analysis of raw reads (Figure 1 [24]). scRNA-seq represents a cutting-edge technique enabling transcriptomic profiling at single-cell resolution, thereby uncovering cellular heterogeneity and cell type-specific expression patterns critical for understanding developmental processes and stress responses [25]. While microarrays and ESTs were early pioneers in transcriptomic analysis, RNA-seq surpasses these technologies with superior accuracy, dynamic range, and discovery capabilities. Each platform exhibits distinct technical strengths and limitations (Table 1), necessitating a careful consideration of research objectives when selecting appropriate methods for gene expression profiling.

### 2.2. Application of Transcriptomics in Plant Stress Resistance Research

Abiotic stress can trigger plants to make alterations at the physiological, morphological, and molecular cellular levels to adapt to unfavorable living environments. Integrated multi-omics analyses reveal that these adaptations involve phosphorylation-mediated signaling cascades and redox homeostasis [38]. Investigating plants’ response mechanisms to various abiotic stresses enables the screening of differentially expressed genes that respond to stress factors, thereby uncovering the relationship between key functional genes and stress resistance. CRISPR-based validation confirms that manipulating these genes enhances the osmotic adjustment capacity [39]. Transcriptomics, which provides a comprehensive study of gene function and structure, can quickly predict defense related factors involved in stress responses. Single-nucleus RNA sequencing now resolves cell type-specific transcriptional reprogramming under stress [40]. This is of great significance for understanding the stress resistance mechanisms of plants and enhancing their capabilities. Machine learning models integrating transcriptomic data accelerate the design of resilient crops [41].

Transcriptomics encompasses a wide range of research aspects, including transcriptome sequencing and analysis, the detection of low-abundance transcripts, the discovery of polymorphic markers, the in-depth exploration of new genes, transcriptome mapping, the identification of gene families, the regulation of alternative splicing, and evolutionary analysis. It has been extensively applied in stress resistance studies of various crops (Table 2). Through transcriptomic analysis, researchers can gain a more comprehensive and in-depth understanding of how plants respond to stress at the gene expression level, which lays a solid foundation for breeding stress-resistant crop varieties.

## 3. Metabolomics

Metabolomics, originally coined as “metabonomics” by Nicholson and Fiehn, is defined as the systematic study of small-molecule metabolites (<1 kDa) within biological systems, including organisms, tissues, and cells [51]. As a cornerstone of systems biology, metabolomics captures the functional readout of cellular processes by quantifying dynamic metabolite profiles. Unlike other omics disciplines, metabolomics directly reflects the integrated effects of genetic and environmental factors, serving as a direct phenotypic readout of biological states. Even subtle genomic or proteomic changes can be amplified and detected at the metabolomic level, making it a powerful tool for uncovering stress-responsive biomarkers [52]. A key advantage lies in its hypothesis-free nature, enabling metabolite profiling across diverse species without prior biochemical or genetic knowledge. This versatility has propelled its application in plant science, where it has been instrumental in deciphering metabolic reprogramming under abiotic stress [53,54].

### 3.1. Characteristics of Metabolomic Research Techniques

Current metabolomic workflows rely on four primary analytical platforms, namely nuclear magnetic resonance (NMR), gas chromatography–mass spectrometry (GC-MS), liquid chromatography–mass spectrometry (LC-MS), and capillary electrophoresis–mass spectrometry (CE-MS). NMR spectroscopy, one of the earliest metabolomic tools, detects nuclear spin transitions induced by electromagnetic fields, providing structural information of metabolites with minimal sample preparation [55]. However, its application is limited by low sensitivity and inability to detect trace metabolites. GC-MS excels in profiling thermally stable, volatile metabolites through chemical derivatization, offering high resolution and reproducibility [56]. Conversely, LC-MS eliminates the need for derivatization and thermal stability, making it suitable for non-volatile and thermally labile compounds such as lipids and phenolic acids [57]. CE-MS separates metabolites based on the charge-to-mass ratio in an electric field, enabling an efficient analysis of highly polar and ionic compounds like amino acids and organic acids [58]. Each platform exhibits distinct analytical capabilities: NMR provides structural insights, GC-MS excels in volatile metabolite profiling, LC-MS offers broad coverage of polar/non-polar analytes, and CE-MS specializes in charged molecules. Table 3 summarizes their technical performance across key metrics, guiding researchers to select appropriate methods based on analyte properties and experimental objectives.

### 3.2. Application of Metabolomics in Plant Stress Resistance

Plant metabolites, as the end products of gene and protein function, are classified into primary and secondary metabolites. Primary metabolites underpin fundamental growth and developmental processes, while secondary metabolites play critical roles in signal transduction, plant–environment interactions, and stress defense mechanisms. These metabolites are particularly crucial in abiotic stress responses, where their dynamic accumulation patterns reflect integrated genetic and environmental cues. Changes in metabolite profiles under stress directly mirror cellular physiological states and biochemical adaptations, making metabolomics a powerful tool for phenotype-to-genotype mapping. By profiling metabolite accumulation across species, tissues, and stress conditions, researchers can identify stress-specific biomarkers and uncover metabolic signatures associated with stress resilience. Recent applications of metabolomics have unraveled key regulatory networks in plants. Table 4 summarizes representative metabolite changes and their functional implications in major crops, highlighting the utility of metabolomics in dissecting stress adaptation mechanisms at the biochemical level.

The accumulation of stress-induced metabolites serves as a hallmark of plant adaptive responses. For example, upregulated tryptophan, threonine, and raffinose in maize under heat stress likely enhance osmoprotection and stabilize heat-denatured proteins through compatible solute accumulation [67]. In wheat, elevated N-based amino acids and ABA under similar conditions reinforce antioxidant defenses and stomatal regulation [68]. Cold-stressed maize exhibits increased L-lysine and L-glutamine, which not only provides nitrogen sources for proline synthesis but also directly stabilizes protein structures as osmoprotectants. L-phenylalanine, a hub in phenylpropanoid metabolism, promotes the biosynthesis of lignin and ferulic acid derivatives [69]. The drought-induced accumulation of 1-aminocyclopropane-1-carboxylic acid (ACC) and putrescine in wheat facilitates ethylene biosynthesis and polyamine-mediated ROS scavenging [73], while salt-stressed barley shows enriched aminoacyl-tRNA and glycine metabolism to sustain protein synthesis and osmotic adjustment [76]. These upregulated metabolites collectively orchestrate stress adaptation through osmotic regulation, redox balance, and signaling reinforcement.

Conversely, the downregulation of specific metabolites under stress conditions provides critical insights into metabolic trade-offs and adaptive resource allocation. For instance, decreased chlorophyll a and glutathione (GSH) levels in maize under heat stress likely reflect impaired photosynthetic efficiency and compromised antioxidant defenses, necessitating alternative energy conservation strategies [81]. Similarly, reductions in fructose-6-phosphate and phosphatidylcholine during cold stress suggest adjustments in glycolytic flux and membrane lipid remodeling to maintain cellular homeostasis [82]. Drought-induced declines in citric acid and malic acid in wheat may indicate suppression of the tricarboxylic acid (TCA) cycle to redirect carbon resources toward osmolyte synthesis [83], while diminished riboflavin and ascorbic acid under salt stress could exacerbate oxidative damage by weakening ROS scavenging capacity [84]. In heavy metal-stressed rice, reduced glutamine and succinic acid levels potentially disrupt nitrogen assimilation and mitochondrial energy metabolism, highlighting metabolic bottlenecks in detoxification pathways [85]. These coordinated downregulations underscore the dynamic rebalancing of metabolic networks to prioritize stress-responsive pathways while conserving resources for survival-critical functions.

## 4. Studies on Transcriptomics and Metabolomics of Plant Responses to Different Abiotic Stresses

Abiotic stressors, including high temperature, cold, drought, flooding, salinity, and heavy metal stress, are prevalent in nature. These adverse environmental conditions impose significant constraints on plant growth and development, invariably affecting crop yield and quality [86]. To counteract abiotic stress, plants have evolved active defense mechanisms. These involve intricate reaction mechanisms encompassing multiple genes, signaling pathways, and metabolic processes [87]. Transcriptomics has the capacity to uncover differential gene expression patterns and intricate regulatory networks under diverse conditions. However, precisely pinpointing key genes, pathways, and regulatory genes remains a challenge. On the other hand, metabolites, which are the end products of gene transcription and protein modification, can be profiled through metabolomics. This helps to elucidate the changes that organisms undergo under genetic or environmental influences. Nevertheless, given the vast and complex nature of plant metabolomes, metabolomic data can only provide partial coverage. Consequently, integrating transcriptomics and metabolomics offers a comprehensive “cause-to-effect” research approach. By simultaneously exploring biological phenomena in plants from these two perspectives, the two techniques can validate and complement each other. This integrated approach is crucial for developing a systematic and comprehensive understanding of plant information transmission processes and the mechanisms underlying functional formation. In recent years, the combined analysis of transcriptomics and metabolomics has emerged as a powerful tool. It has been instrumental in identifying key genes and metabolites associated with plant responses to abiotic stress. Additionally, it has significantly contributed to exploring the molecular mechanisms governing plant growth, development, and stress responses.

### 4.1. Studies on Transcriptomics and Metabolomics of Plant Responses to Temperature Stress

**Figure 2:**
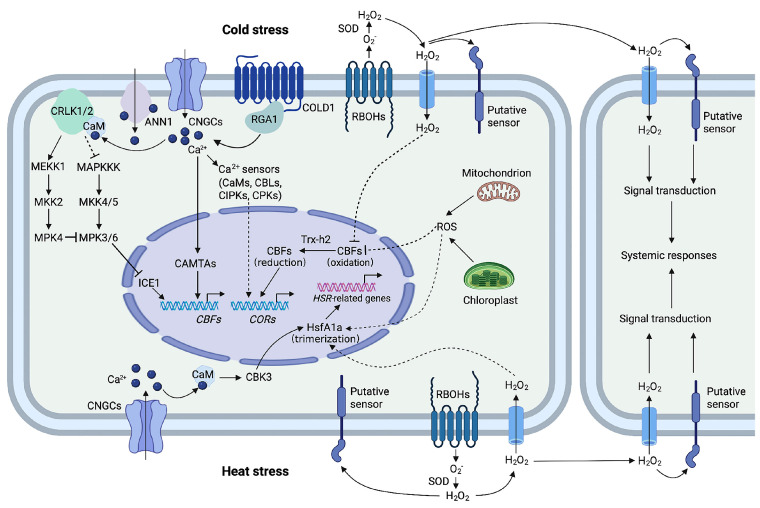
Roles of ROS and Ca^2+^ in plant responses to temperature stress (Ca^2+^ and ROS are important molecules involved in regulating temperature stress responses. Cold and heat-induced Ca^2+^ signatures may be decoded by Ca^2+^ sensors or Ca^2+^-related proteins and thus regulate COR and HSR gene expression. Moreover, ROS regulate CSR and HSR by modulating CBFs and HsfA1a, respectively. ROS may also play an important role in temperature stress-triggered systemic responses. Arrows indicate positive regulation and T-bars indicate negative regulation).

Temperature is a crucial environmental factor for plant growth and development. Only under suitable temperature conditions can plants conduct normal material transport and energy exchange. When exposed to temperature stress, plants experience a series of physiological changes. These changes can lead to irreversible damage to cellular homeostasis, the degradation of functional proteins, and the disruption of metabolic pathways, which in turn affect crop quality and yield and may even cause plant death. Yang et al. proposed that upon sensing temperature stress, plants rapidly initiate a complex signal transduction pathway specific to heat or cold stress [88]. This process involves multiple components, such as Ca^2+^, reactive oxygen species (ROS), and various regulators. These components regulate the expression of temperature-responsive genes and the metabolism of temperature-responsive proteins, as illustrated in Figure 2 [88].

#### 4.1.1. Heat Stress

Due to global warming, extreme high-temperature events are happening much more frequently. Heat stress, as a major abiotic stressor, severely restricts plant growth and reduces crop yields [89]. When confronted with heat stress, plants respond through various means, mainly by regulating antioxidant substances, osmolytes, heat shock proteins, starch metabolism, amino acid levels, and protein structure. Wang et al. employed transcriptome and metabolome analyses to explore the response mechanism of sweet corn to heat stress [90]. Their findings indicated that heat stress affected both phenylpropanoid compounds and photosynthesis-related pathways. Moreover, the expression levels of numerous alkaloids and flavonoid compounds were upregulated. Notably, RNA-dependent RNA polymerase 2, UDP-glucosyltransferase *73C5*, *LOC103633555*, and tetrachloride interactive domain 7 were identified as four key hub genes under heat stress conditions. Hu et al. conducted transcriptome and metabolome analyses on two tall fescue varieties with distinct temperature sensitivities [91]. They discovered that under heat stress, the transcriptional abundance of 12 enzyme-related genes in the carbohydrate metabolic pathway increased substantially. Additionally, the contents of sugars and lipids accumulated progressively with extended heat treatment duration. Wang’s research on two pepper varieties with different heat-tolerance levels, through transcriptome and metabolome analyses, identified 5754 and 5756 differentially expressed genes in heat-tolerant and heat-sensitive varieties, respectively, along with 94 and 108 differentially accumulated metabolites [4]. Specifically, alterations in amino acids, organic acids, flavonoid compounds, and sugars underscored the complex reaction mechanisms underlying pepper heat tolerance. The glutathione metabolic pathway also played a pivotal role in pepper’s response to heat stress. Guo et al. examined the development and starch deposition changes in waxy corn kernels post-pollination under different temperature treatments [92]. Using transcriptome and metabolome analyses, they found that the indole-3-acetic acid (IAA), abscisic acid (ABA), and salicylic acid (SA) signaling pathways underwent significant changes during the heat stability response. The signaling pathways mediated by coenzymes, ABA, and SA were more active in response to heat stress, and these hormones were crucial for grain development. Xiang et al. applied combined transcriptome and metabolome analysis and demonstrated that high-temperature stress significantly decreased the contents of zeatin, salicylic acid, jasmonic acid, and auxin in maize [93]. The upregulation of ARR-B genes enhanced zeatin signaling transduction, thereby improving maize heat tolerance.

#### 4.1.2. Cold Stress

Cold stress poses a significant threat to plants, often resulting in frost damage. This stress not only restricts plant growth and development, which in turn impacts crop quality and yield, but it also influences the distribution of plant species [94]. In response to cold stress, plants initiate a series of changes in hormone signaling, photosynthesis, osmolyte regulation, synthesis, and metabolic pathways, as well as the expression of related genes. Guo et al. investigated the response mechanism of maize to cold stress and found that the content of endogenous abscisic acid (ABA) increased under such conditions [95]. This finding suggests that ABA plays a crucial role, and the identified genes could potentially serve as targets for breeding cold-resistant waxy corn varieties. Xu et al. carried out transcriptome sequencing and metabolomic analysis on tobacco plants subjected to low-temperature stress [96]. They identified 6905 differentially expressed genes (DEGs), which were mainly involved in signal transduction, carbohydrate metabolism, and phenylpropanoid biosynthesis. Additionally, 35 protective metabolites were detected. These metabolites, including amino acids, carbohydrates, tricarboxylic acid (TCA) cycle intermediates, and phenylpropanoid-related substances, participated in the cold stress response. Li et al. compared the physiological activities of different pumpkin inbred lines under varying temperature conditions [97]. Through transcriptome and metabolome analyses, they found that cold stress significantly elevated the levels of malondialdehyde, relative conductivity, soluble proteins, sugar content, and catalase activity. Moreover, cold stress activated the transcription of genes involved in plant hormone signal transduction pathways and transcription factor families such as AP2/ERF, bHLH, WRKY, MYB, and HSF.

### 4.2. Transcriptomic and Metabolomic Studies of Plant Responses to Water Stress

Water is an indispensable environmental factor for plant life. Its availability is the key determinant of plant growth, yield, and quality formation. Water deficiency delays plant development, markedly inhibiting seed germination and seedling growth. In extreme cases, it can cause substantial crop yield losses. Conversely, excessive water reduces plant root activity, impeding normal respiration. Under hypoxic stress resulting from waterlogging, plant photosynthesis declines, severely restricting related physiological metabolism and growth and ultimately leading to cell death [98]. Studies indicate that plants can adapt to adverse water-stressed conditions by modifying their physicochemical properties. The principal molecular mechanism underlying plants’ response to water stress involves cells perceiving and transmitting stress signals. This process regulates diverse metabolic and signal transduction pathways, prompting responses at the transcriptional and translational levels and thereby altering the expression of relevant genes [99].

#### 4.2.1. Drought Stress

Drought ranks among the most critical factors constraining plant production and jeopardizing global food security. When plants are exposed to drought stress, they undergo numerous physiological alterations. Under such conditions, crops maintain the osmotic pressure equilibrium across cell membranes by leveraging small molecules like sugars and amino acids. This mechanism is crucial for plants to cope with drought stress, modulate their resistance, and regulate growth [100]. Mathan et al. discovered that drought stress led to an increase in the sucrose content within the leaves, root tissues, and phloem sap of Oryza sativa L. varieties [101]. This elevation in sucrose levels helps maintain sugar homeostasis within rice plants, enabling them to better withstand drought stress. Li et al. employed a combined transcriptome and metabolome analysis and demonstrated that drought stress decreased the activity of sucrose synthase genes sus1 and SH-1 [102]. This reduction inhibited the conversion of sucrose to UDP-glucose, ultimately resulting in a decline in pollen vitality. Wei et al. conducted a transcriptome analysis on two drought-tolerant and two drought-sensitive maize lines [103]. Through this research, they identified the drought-resistant gene *ZmbHLH124*. This gene has the ability to activate the transcription of the drought-related *ZmDREB2A* gene, thereby enhancing maize’s tolerance to drought. Ackah et al. performed a metabolome analysis on the mulberry variety Yu-711 under drought stress [74]. They found significant changes in the levels of total lipids, galactolipids, and phospholipids, which accounted for 48% of the total differentially expressed metabolites. Notably, fatty acyl-based substances decreased by 73.6%. Additionally, other metabolites such as polyphenols (including flavonoids and cinnamic acid), organic acids (e.g., amino acids), carbohydrates, phenyl compounds, and organic heterocyclic substances also exhibited dynamic responses to drought stress. These findings suggest that mulberry copes with drought stress through the differential accumulation of metabolites. Li et al. subjected two maize inbred lines, si287 (drought-tolerant) and X178 (drought-sensitive), to drought stress [104]. Using transcriptome and metabolome analysis techniques, they detected significant differences in pathways related to glycolysis/gluconeogenesis, flavonoid biosynthesis, starch and sucrose metabolism, and amino acid biosynthesis. Proline, tryptophan, and phenylalanine were identified as key amino acids for maize in responding to drought stress. Abscisic acid (ABA) is a hormone that exerts a profound influence on plants’ response to drought stress [105]. Plants can accumulate substantial amounts of ABA in their tissues. This accumulation triggers the ABA signal transduction pathway, which induces the expression of stress-related genes and ultimately enhances plant drought tolerance. Xiong et al. conducted a transcriptome analysis of soybean plants under drought stress [106]. They found that 26 GmPP2A-B00 members within the soybean PP2A-B00 family genes responded to drought. Given that protein phosphatase 2A plays a vital role in regulating cellular reactive oxygen species (ROS) signaling, these results imply that GmPP2A-B genes can promote soybean drought resistance by modulating ROS signal transduction. Wei et al. utilized liquid chromatography–mass spectrometry (LC-MS) to investigate the metabolomic changes in safflower (*Carthamus tinctorius* L.) under drought stress [107]. The analysis identified 359 and 209 differential metabolites in varieties PI401477 and PI560169, respectively. Moreover, three metabolites, namely galactitol, neoxanthine, and arbutin, were found to be associated with drought tolerance.

#### 4.2.2. Waterlogging Stress

Waterlogging stress has emerged as a major abiotic stressor that significantly impacts crop growth, development, and yield. It affects approximately 12% of the global land area and can cause up to a 20% reduction in crop yields. The most immediate consequence of waterlogging stress on plants is the disruption of aerobic respiration, which in turn restricts a range of essential processes, including energy metabolism, growth, and physiological metabolism [108]. Plants have evolved various strategies to adapt to such adverse environments. They regulate their morphological structure, energy metabolism, endogenous hormone biosynthesis, and signal transduction pathways [109]. Under waterlogging conditions, the oxygen supply in the soil is severely limited. Hypoxia not only stimulates but also exacerbates the accumulation of reactive oxygen species (ROS), ultimately resulting in cell death and plant senescence. To counteract oxidative stress, plants have developed an antioxidant defense system. This system helps maintain the dynamic balance of ROS in the body, alleviates ROS-induced cellular damage, and promotes cell survival [110]. Luan et al. compared the waterlogging tolerance of barley varieties Franklin and TX9425 through transcriptome analysis [111]. They identified 124 differentially expressed genes (DEGs) involved in ROS scavenging. These genes are associated with the synthesis of glutathione S-transferase, peroxidase, catalase, and L-ascorbate peroxidase, with most of them being related to peroxidase. Their findings also highlighted the important role of alcohol dehydrogenase in plants’ response to waterlogging stress. Feng et al. conducted a comparative study on the waterlogging tolerance of two sweet corn varieties, D120 and D81 [112]. Transcriptome analysis revealed 2492 and 2351 differentially expressed genes in these two varieties under waterlogging stress. In the waterlogging-tolerant D120 genotype, genes related to ROS balance were highly expressed, enhancing the plant’s ROS scavenging ability. Moreover, the ZmERF055 gene located on chromosome 9 was identified, which can enhance plant waterlogging tolerance and maintain ROS balance. Hong et al. subjected two rapeseed inbred lines, G230 and G218, to waterlogging stress and performed a combined transcriptome and metabolome analysis [113]. The results showed that the differentially expressed genes and metabolites were mainly enriched in metabolic pathways, secondary metabolite biosynthesis, flavonoid biosynthesis, and vitamin B6 metabolism. Multiple studies have demonstrated that plant hormones play a crucial role in plants’ response to waterlogging stress. Ethylene, for example, is essential for plants to adapt to hypoxia and metabolic changes during flooding. It enables plants to adapt to hypoxic environments by enhancing the stability of the ethylene response factor family VII (ERF-VIIs) [114,115]. Yuan Cheng’s transcriptome and metabolome analysis of winter wheat grains under flooding stress indicated that regulating the expression of ethylene, abscisic acid, and jasmonic acid-related synthesis genes can improve crop waterlogging tolerance [116]. Pathways such as the ascorbate–glutathione cycle and sugar metabolism work together to resist flooding stress. Sreeratree et al.’s research also confirmed the important role of ethylene and jasmonic acid in enhancing waterlogging tolerance. Wu et al. subjected CM37 and cmh15 seedlings to waterlogging stress and analyzed the transcriptome sequencing data [117]. They found that the differentially expressed genes were mainly enriched in photosynthesis, photosystem pathways, and glycolysis/gluconeogenesis pathways. Owusu et al. used transcriptome analysis to identify several key genes for cotton waterlogging tolerance, including PER1, PRX52, PER64, ADH, PDC, MT1, XTH, and SUS [118]. These genes are closely related to the antioxidant system. Additionally, transcription factors such as WRKY, AP2/ERF, and MYB play a vital role in the waterlogging tolerance mechanism. Metabolome analysis further revealed that waterlogging stress significantly induced pathways such as phenylpropanoid biosynthesis, galacturonate synthesis, valine, leucine, and isoleucine biosynthesis, purine metabolism, and galactose metabolism. The accumulation of these amino acids significantly contributes to enhancing plant waterlogging tolerance. In conclusion, the regulatory mechanisms underlying plants’ response to waterlogging stress are highly intricate. Integrating transcriptome and metabolome analysis offers a powerful and efficient approach for identifying waterlogging-tolerance genes and exploring related metabolites.

### 4.3. Research on Transcriptomics and Metabolomics of Plant Responses to Heavy Metal Stress

Heavy metal stress stands out as one of the most detrimental abiotic stresses, exerting a profound impact on the growth, development, yield, and quality of crops. This stress factor presents a significant hurdle to the sustainable development of agriculture. Heavy metals encompass a category of biologically toxic metals and metalloids, including cadmium (Cd), lead (Pb), mercury (Hg), and arsenic (As), among others. Even at trace levels, these substances can disrupt the physiological and morphological processes in plants. They negatively affect plant growth via various mechanisms such as osmotic stress, ion imbalance, oxidative stress, membrane disruption, cellular toxicity, and metabolic derangements. In severe cases, heavy metal exposure can ultimately lead to the death of plants [119].

Studying plant responses to heavy metal stress using transcriptomics and metabolomics is pivotal to comprehensively dissect the intricate regulatory networks associated with heavy metal stress responses. This approach serves as a crucial means for screening and breeding heavy metal-tolerant plants. Zhou et al. delved into the response mechanism of wheat under cadmium stress [120]. Through transcriptome analysis, they identified 1561 differentially expressed genes (DEGs) in L17 and 297 DEGs in H17. These genes were predominantly involved in terpenoid backbone biosynthesis, phenylalanine metabolism, photosynthesis, ABC transporters, and glutathione metabolism in the context of cadmium stress. Dubey et al. examined rice roots under chromium stress and found 1138 upregulated genes and 1610 downregulated genes [5]. The majority of the genes that exhibited differential expression under the two chromium stress conditions were related to glutathione metabolism, transport, and signal transduction pathways, highlighting the critical role of glutathione in the detoxification process during chromium stress. Mwamba et al. utilized metabolomics techniques to explore changes in metabolite contents in Brassica napus under different cadmium stress conditions [121]. They observed that lignin was highly expressed in high-cadmium-accumulating Brassica napus, indicating its potential to impede cadmium entry into plants. Additionally, plant sterols, monoterpenes, and carotenoids were induced by cadmium. In cadmium-tolerant Brassica napus, unsaturated fatty acids, lipoproteins, and glycerophospholipids accumulated significantly, and inositol-derived signaling metabolites were induced, which could rapidly trigger detoxification mechanisms in plants. Lai et al. conducted a metabolomic analysis and demonstrated that under the combined induction of uranium and cadmium, the expression of antioxidant substances in purple sweet potato cells increased substantially, thereby enhancing the plant’s tolerance to heavy metals [80]. Wei et al. employed metabolomics and transcriptome sequencing to investigate the metabolic and transcriptional response mechanisms of Kochia scoparia to cadmium stress [122]. Cadmium stress affected the accumulation and transport of cadmium in plants, increased the content of soluble sugars, enhanced the activities of ascorbate peroxidase and peroxidase, and decreased the activity of superoxide dismutase, ultimately influencing plant growth and development. Glutathione metabolism and lignin biosynthesis were identified as key metabolic pathways. These studies suggest that analyzing and comparing the differences in transcription levels between tolerant and sensitive genotypes under heavy metal stress from a transcriptomic perspective can help identify stress tolerance-related genes. Simultaneously, from a metabolomic perspective, plant responses to heavy metal stress mainly involve reducing heavy metal absorption, chelating heavy metals, activating antioxidant defenses, and scavenging free radicals [123].

### 4.4. Research on Transcriptomics and Metabolomics of Plant Response to Salt Stress

Soil salinity stress represents a major constraint on the stable yield and income of crops. It poses a substantial threat to agricultural production and is one of the primary abiotic stresses that plants encounter [124]. High-salt conditions induce osmotic stress in plants, resulting in water deficits, physiological imbalances, and metabolic disruptions [125]. Additionally, salt stress can lead to ion toxicity, causing the excessive accumulation of reactive oxygen species (ROS) within the plant. This, in turn, inflicts oxidative damage on organelles and membrane components, directly impacts proteins and photosynthesis, inhibits plant growth, and may ultimately culminate in plant death. To mitigate the adverse effects of salt stress, plants have evolved several strategies. They can enhance their salt tolerance through osmotic adjustment, maintaining ion balance, scavenging ROS, and hormonal regulation [126].

The application of transcriptomics and metabolomics in studying the response mechanisms of crops to salt stress, as well as in identifying key genes and metabolites that regulate biological processes, has been widely carried out in plants such as maize, rice, barley, soybean, and tobacco. Xu et al. investigated the adaptive response of oat cultivars to salt stress. Metabolomic analysis uncovered 201 metabolites, including sugars, amino acids, organic acids, and secondary metabolites. Salt stress disrupted the biosynthesis, energy consumption, and sugar metabolism of BY2 and BY5. The distinct defense capabilities of these two oat cultivars against salt stress were attributed to differences in their energy consumption strategies, energy material synthesis, and root ion transport [127]. Lu et al. conducted transcriptomic and metabolomic analyses on grapevines under saline–alkali stress. The stress induced signal transduction and metabolic processes, accompanied by significant increases in the contents of ascorbic acid, glutathione, most phenolic acids, flavonoids, and alkaloids. The biosynthetic pathway of flavonoids plays a vital role in the response of grapes to salt stress. The results demonstrated that the plant’s response to saline–alkali stress is closely associated with the antioxidant system’s ability to scavenge reactive oxygen species (ROS) [128]. Han et al. performed metabolomic and transcriptomic analyses on cotton under salt stress treatment, revealing that salt stress increased the contents of amino acids, sugars, and abscisic acid (ABA) while reducing those of vitamins and terpene compounds. Among them, the accumulation of cysteine, ABA, isopentenyl adenine-7-N-glucoside, and tulipose is crucial for the salt tolerance mechanism of cotton [129]. Jin et al. studied the salt tolerance mechanisms of three soybean cultivars (JD19, LH3, and LD2) with different salt tolerances. Transcriptomic analysis showed that compared with LD2, salt stress enhanced antioxidant metabolism, stress response metabolism, glycine metabolism, serine metabolism, and gene expression related to transcription and translation in JD19 and LH3. Metabolomic analysis revealed that amino acid metabolism and the tricarboxylic acid (TCA) cycle are important metabolic pathways for soybeans to cope with salt stress [130]. Shu et al. investigated the mechanism of Brassica napus’s response to salt stress, indicating that abscisic acid and jasmonic acid are key factors in the process of responding to salt stress. Meanwhile, some metabolites, such as N-acetyl-5-hydroxytryptamine, L-cysteine, and L-(+)-arginine, play a crucial role in maintaining ROS balance [131]. Ma et al. found that Arabidopsis seedlings were more tolerant to salt stress in the light than in the dark. The study showed that photoreceptors phytochrome A (phyA) and phyB were involved in this tolerance mechanism. phyA and phyB physically interacted with SALT OVERLY SENSITIVE2 (SOS2) in both the cell membrane and the nucleus, enhancing the activity of the SOS2 kinase in the light. Meanwhile, SOS2 directly phosphorylates PIF1 and PIF3 in the nucleus to promote salt tolerance in plants [132]. Gao et al. demonstrated that nitric oxide (NO) could participate in mediating plant physiological responses under salt stress. The exogenous application of the NO donor, sodium nitroprusside (SNP), increased the fresh weight, shoot and root elongation, and reduced electrolyte leakage and malondialdehyde (MDA) content in N. tangutorum seedlings under salt stress. Meanwhile, SNP activated the ascorbate–glutathione (AsA-GSH) cycle to scavenge reactive oxygen species (ROS) and alleviate the oxidative damage caused by salt stress [133]. Wang employed liquid chromatography (LC) combined with electrospray ionization time-of-flight mass spectrometry (ESI-TOF-MS) to analyze metabolites in the cotyledons and roots of the castor plant (Ricinus communis) under salt stress. The results showed that the metabolites in the cotyledons and roots under salt stress differed significantly. In addition, KEGG pathway enrichment analyses indicated that flavonoid and flavonol biosynthesis, pantothenic acid and CoA biosynthesis, the citric acid cycle, and carotenoid biosynthesis were common metabolic pathways in response to salt stress [134]. These results suggest that salt stress can induce the establishment of plant defense signaling networks by influencing the homeostasis of plant hormones.

### 4.5. Emerging Insights into Other Abiotic Stressors

While the primary focus has been on thermal, water, salt, and heavy metal stresses, plants also face diverse challenges from additional environmental factors. Recent research utilizing multi-omics techniques has started to reveal the molecular mechanisms behind these stressors. For example, excessive light or UV-B exposure induces ROS accumulation and DNA damage. Studies have shown that in response to high light stress, plants initiate a series of signal transduction processes, which trigger various physiological and biochemical reactions to mitigate the harmful effects of high light (such as photodamage and photoinhibition). The protective mechanisms of plants against light stress include scavenging chloroplastic reactive oxygen species, chloroplast and stomatal movement, and anthocyanin synthesis [135]. ROS bursts triggered by multiple stressors are mitigated by coordinated gene–metabolite responses. In maize, combined stress upregulates ZmSOD4 and ZmAPX6, while metabolomics reveals glutathione (GSH) accumulation [67]. Studies have shown that phosphorus (P) deficiency in rice induces the OsPHR2-mediated transcriptional reprogramming of phosphate transporters (OsPTs), while metabolomics identifies an increased synthesis of organic acids (malate, citrate) to enhance P solubilization [136]. Iron (Fe) excess in barley activates HvNRAMP3 transporters and vacuolar sequestration genes, with a concurrent accumulation of Fe-chelating phenolics [137]. Air pollutants (e.g, ozone O_3_ and PM_2.5_) directly impair plant physiology by suppressing key photosynthesis genes (RBCS, RCA), inducing oxidative stress (NADPH oxidase-mediated ROS activation), and disrupting stomatal function [138]. Concurrently, studies demonstrate that hyperaccumulator plants (e.g, Brassica juncea for heavy metal enrichment) and plant–microbe symbiotic systems (rhizosphere microbiota degrading PAHs) effectively enhance environmental remediation [139]. Emerging technologies, including nanomaterials and integrated multi-omics analyses, provide novel strategies for resistance breeding and pollution control [140].

## 5. Perspectives and Conclusions

In summary, as integral components of systems biology, transcriptomics and metabolomics have furnished reliable research tools. They enable the rapid prediction of stress-related defense factors, the revelation of the relationships among metabolic pathways, signal transduction, and defense responses, the identification of metabolite types and their changing patterns, and the uncovering of diverse changes in plants under abiotic stress. These techniques substantially contribute to enhancing our comprehension of plant stress resistance and its underlying mechanisms. Notably, the integrated analysis of transcriptomics and metabolomics assumes a pivotal role in clarifying the genetic basis of plants’ response and adaptation to abiotic stress. It promotes the cultivation of stress-resistant varieties and bolsters the stable yield of crops. Although some genes associated with plant stress-resistant metabolic pathways have been cloned and their molecular mechanisms are being gradually elucidated, our understanding of plant stress resistance is still circumscribed. Therefore, further research is imperative to explore these pathways and other synergistic effects.

Plants’ response to adversity stress represents a highly intricate process. It encompasses the perception of stress signals, signal transduction, and the activation of defense mechanisms, all of which are underpinned by elaborate metabolic networks. In natural settings, when plants are confronted with multiple types of stresses, cross-effects often occur. Gaining insights into the specific as well as the cross-shared signal transduction and metabolic pathways in plants, along with devising efficient methods to mine functional genes and accurately identify metabolites, are of utmost importance in researching plant stress resistance genes. Furthermore, numerous beneficial soil microorganisms within the rhizosphere microbiota have been found to enhance plants’ resistance to abiotic stresses. However, the underlying molecular mechanisms remain largely uncharted. Deciphering the molecular mechanisms governing the protective effects of these beneficial microorganisms could potentially open up new strategies. These strategies may improve the effectiveness of genetic, chemical, and microbiological approaches in enhancing plant stress tolerance.

Currently, the remarkable progress of high-throughput technologies has rendered data acquisition in transcriptomics and metabolomics more cost-effective and efficient. This enables researchers to delve deeper into the molecular regulation mechanisms and metabolic regulatory networks of various crops when responding to adversity stress. Nevertheless, there is a pressing need for further exploration to effectively screen and utilize the core data amidst the vast amount of information. Data analysis in transcriptomics and metabolomics is an intricate process. It involves the application of numerous analytical software tools to guarantee the accuracy and reliability of the results. Examples of such tools include OmicsSuite, MetaboAnalyst, MetaCoreTM, InCroMAP, MetFrag, etc. [141,142]. These software tools offer researchers powerful means to handle and analyze complex transcriptional and metabolomic data. However, they also have certain limitations. These mainly encompass insufficient data processing capabilities, a lack of comprehensive bioinformatics support, the absence of standard products and database products, and a high cost associated with high-throughput analyses. As a result, continuous exploration and development are essential to better support research in resistance breeding. Although the integrated analysis of transcriptomics, metabolomics, and genome-wide association studies is currently being carried out in a relatively in-depth and systematic manner, there are still issues such as inadequate integration with other omics technologies and insufficient in-depth data mining. Hence, it is necessary to further integrate transcriptomics, metabolomics, and other omics technologies and develop more efficient bioinformatics analysis techniques to meet the requirements of data collection, storage, and computational analysis. Multi-omics and multi-omics integration technologies offer a novel breeding approach for abiotic stress breeding. By integrating data from various omics platforms, they can enhance the comprehensive and systematic understanding of abiotic stress. This allows for the identification of key regulatory networks, biomarkers, and candidate genes that can serve as targets for breeding programs, thereby facilitating the realization of precision agriculture. Multi-omics technology strategies establish a connection between the genotype and the phenotype of a plant. Unveiling the overall mechanism of how plants respond to abiotic stress, from genes to traits, will provide a solid foundation for a better elucidation of the molecular mechanisms underlying biological processes. With the continuous advancement of multi-omics technologies, more resistance-related genes will be discovered, enabling a more comprehensive revelation of the essence of plant stress resistance. Simultaneously, the big data obtained from multiple omics layers, in combination with advanced bioinformatics, can be employed for predictive modeling and precision breeding. This will contribute to the more precise breeding of resilient varieties and the promotion of sustainable agriculture, thus ensuring global food security.

## Figures and Tables

**Table 1:** Comparative analysis of transcriptomic technologies.

Technology	Theory	Advantage	Limitation	Examples
Microassay [26]	Hybrid	1. Fast speed 2. Low cost 3. Simple sample preparation 4. Flexible analysis range	1. The sensitivity for detecting low-expression genes is insufficient 2. The sensitivity of hybridization technology is limited 3. The prerequisite work requires a high level of foundation 4. It is difficult to detect abnormal transcription products	Microarray technology was used for the expression analysis of genes in Arabidopsis thaliana responding to salt stress, and salt-tolerant-related genes were screened by fluorescence signal differences [27].
EST [28]	Sanger	1. Detection range is wide 2. Accuracy is high 3. Improves the efficiency of gene isolation	1. The sequencing read length is short 2. The error rate is high 3. The sequencing throughput is low	Multiple gene fragments encoding protease inhibitors were identified through EST technology, providing a molecular basis for insect-resistant breeding [29].
SAGE [30]	Sanger	1. High-throughput detection 2. Can quantitatively evaluate gene expression levels 3. Gain a comprehensive understanding of gene expression regulation mechanisms	1. High cost 2. Complex data processing 3. Relying on known gene databases, there are certain limitations in identifying unknown genes	The significant upregulation of ABA synthesis-related genes was discovered using SAGE technology, revealing the critical role of ABA in drought responses [31].
MPSS [32]	Sanger	1. High throughput 2. Quantitatively display the expression of genes within cells	1. High cost 2. Complex operation 3. Difficulties in bioinformatics processing	In yeast transcriptome research, MPSS (Massively Parallel Signature Sequencing) technology is used for genome-wide quantitative analysis of gene expression [33].
RNA sequencing [34]	High-throughput sequencing	1. High throughput 2. High accuracy 3. Wide detection range 4. Low cost	1. The sample preparation is cumbersome 2. It cannot reveal the heterogeneity of expression among single cells 3. The bioinformatics analysis tools are limited	RNA-sequencing analysis revealed that the expression patterns of genes associated with translation, membrane, and oxidoreductase activity pathways were altered under drought stress [35].
scRNA-seq [36]	High-throughput sequencing	1. High accuracy and specificity 2. Clarify cell function and localization	1. High requirements for sample quality 2. High cost 3. Difficulties in data analysis/interpretation	Single-cell analysis of Arabidopsis root tips reveals specific transcriptional responses of epidermal and cortical cells under salt stress [37].

**Table 2:** The list of key genes of plants in response to abiotic stresses.

Gene	Crops	Abiotic Stress	Gene Function	References
*OsWRKY87*	Rice	Drought, salt stress	*OsWRKY87* functions as a transcriptional activator	[42]
*OsSEH1*	Rice	Cold stress	*OsSEH1* regulates the expression and metabolite accumulation of genes related to phenylpropanoid and flavonoid biosynthesis, mediating ABA expression levels in response to cold stress	[43]
*OsCSLD4*	Rice	Salt stress	*OsCSLD4*, a cell wall polysaccharide synthase, responds to salt stress through ABA-induced osmotic stress	[44]
*OsNAC5*	Rice	Drought stress, cold stress	It enhances stress tolerance by upregulating the expression of the *OsLEA3* gene	[45]
*ZmHsf01*	Maize	Heat stress	Plays an important role in heat shock signal transduction and downstream gene expression	[46]
*ZmNAC3*	Maize	Cold stress, salt stress	*ZmNAC3* encodes a nuclear-targeted protein with a highly conserved NAC domain at its N-terminus	[47]
*ZmICE1*	Maize	Cold stress	*ZmICE1* regulates the expression of the *DREB1* gene, inhibits the expression of *ZmAS*, and reduces Glu/Asn biosynthesis, thus alleviating the production of reactive oxygen species	[48]
*TdSHN1*	Wheat	Heavy metal stress	Enhances cadmium tolerance by increasing the activity of superoxide dismutase and catalase	[49]
*ZmCAO1*	Maize	Waterlogging stress	Mutation of *ZmCAO1* leads to the downregulation of key photosynthetic genes, increased reactive oxygen species, and sensitivity to waterlogging	[50]

**Table 3:** Characteristics of metabolomic technologies.

Technology	Targets	Advantage	Limitation	Examples
liNMR	Most compounds in metabolites	1. Small sample size required 2. No sample preprocessing required 3. Accurate provision of metabolite structure information	1. Low detection sensitivity and resolution 2. Difficult to detect low-abundance metabolites 3. High requirements for sample preparation	Plant holistic metabolic profiling, metabolic flux analysis, in situ/non-destructive metabolic studies [59,60].
1GC-MS	Volatile, gasifiable, or small molecules	1. High-resolution and sensitivity 2. Ability to identify metabolite structures 3. Easy qualitative analysis of metabolites	1. Unable to separate macromolecules 2. Cannot analyze thermally unstable and non-gasifiable substances 3. Complex and time-consuming derivatization preprocessing procedures	Plant primary metabolite analysis, volatile organic compound analysis, fatty acid profiling [61,62].
LC-MS	High boiling point, non-volatile, non-derivatizable macromolecules	1. High detection sensitivity 2. Fast analysis speed 3. Ability to separate metabolites with similar structures	1. Limited database size 2. Limited types of metabolites analyzed 3. Not all metabolites can be accommodated by the same column material	Plant secondary metabolite analysis, lipidomics—phospholipids, glycolipids, phytohormone quantification [63,64].
CE-MS	Trace, complex samples	1. High detection sensitivity 2. Fast analysis speed 3. Small sample size required 4. Wide coverage of metabolites	1. High requirements for equipment and devices 2. Small sample size, poor reproducibility of separation 3. Narrow linear range for quantitative analysis 4. Limited quantitative analysis due to narrow linear range	Highly polar/ionic compound analysis, analysis of minute samples (single-cell metabolomics) [65,66].

**Table 4:** The list of metabolites of plants in response to abiotic stresses.

Abiotic Stress	Crops	Metabolite	Change	References
Heat stress	Maize	Tryptophan, Threonine, Histidine, Raffinose, Galactitol, Lactitol	Upregulated	[67]
Heat stress	Wheat	N-based Amino Acids, ABA, IAA-conjugates	Upregulated	[68]
Cold stress	Maize	Guanosine 30, 50-Cyclic Monophosphate, Sophoroside-7-O-Glucoside, L-Lysine, L-Phenylalanine, L-Glutamine, Shanenol, Feruloyl Tartaric Acid	Upregulated	[69]
Cold stress	Maize	Trans-aconitate, Coumaroyl Hydroxycitrate, Geranyl Glucosyl Rhamnoside Rhamnoside, Caffeoylquinate, Ferroylquinate, (Iso)Vitexin, DIBOA-Glucoside	Upregulated	[70]
Cold stress	Maize	Chlorophyll, Glucose-6-Phosphate Dehydrogenase, Sucrose-to-Starch Ratio	Upregulated	[71]
Cold stress	Canola seed	Amino Acids, Organic Acids, Sugars	Upregulated	[72]
Drought stress	Wheat	1-Aminocyclopropane-1-Carboxylic Acid, Asn, 5-HT, GABA, Cystine, Deoxyuridine, Tryptamine, Putrescine	Upregulated	[73]
Drought stress	Mulberry tree	Galactolipids, Phospholipids, Flavonoids, Cinnamic Acid, Amino Acids, Carbohydrates, Benzenoids, Organic Heterocyclic Compounds	Upregulated	[74]
Drought stress	Barley	Amino Acids, Sugars, Abscisic Acid, Jasmonic Acid, Ferulic Acid	Upregulated	[75]
Salt stress	Barley	Aminoacyl-tRNA Biosynthesis, Glycine, Serine, and Threonine Metabolism, Glyoxylate and Dicarboxylate Metabolism, Porphyrin and Chlorophyll Metabolism	Upregulated	[76]
Salt stress	Wheat	Amino Acids and Derivatives, Flavonoid Compounds, Organic Acids and Derivatives, Nucleotides and Derivatives, Lipids	Upregulated	[77]
Salt stress	Blueberry	Glycine, Malic Acid, Octadecanoic Acid, L-Threonic Acid	Upregulated	[78]
Heavy metal stress	Rice	Lipids, Eicosanoids	Upregulated	[79]
Heavy metal stress	Purple sweet potato	Glutathione, Tryptophan	Upregulated	[80]
Heat stress	Maize	Chlorophyll a, Glutathione (GSH)	Downregulated	[81]
Cold stress	Maize	Fructose-6-phosphate, Phosphatidylcholine	Downregulated	[82]
Drought stress	Wheat	Citric Acid, Malic Acid	egulaDownrted	[83]
Salt stress	Wheat	Riboflavin, Ascorbic Acid	Downregulated	[84]
Heavy metal stress	Rice	Glutamine, Succinic Acid	Downregulated	[85]

## Data Availability

No new data were created or analyzed in this study.
